# Leveraging journal citation-based metrics for enhanced university rankings methodology

**DOI:** 10.3389/frma.2024.1510169

**Published:** 2024-12-18

**Authors:** Ali Ghaddar, Sergio Thoumi, Samer S. Saab

**Affiliations:** ^1^Department of Electrical and Computer Engineering, Lebanese American University, Byblos, Lebanon; ^2^Department of Computer Science and Mathematics, Lebanese American University, Beirut, Lebanon

**Keywords:** ARWU, THE, QS, research assessment, citation metrics, ranking evaluation, citation-based metrics

## Abstract

This paper proposes a novel framework for evaluating research performance in university rankings, utilizing journal citation-based metrics and scholarly output instead of traditional article citation metrics. Through correlation analysis, we compare the proposed metrics with article citation metrics used by prominent ranking systems (THE and QS) and demonstrate significantly higher correlations with established rankings (QS, THE, and ARWU). The proposed metrics exhibit robustness over time and offer a fairer evaluation by emphasizing objective performance and mitigating citation biases. This framework provides institutions with a more accurate benchmarking tool to inform strategic decisions and resource allocation. While acknowledging potential limitations in data availability and the challenge of achieving global consensus, this study contributes to the ongoing discourse on university rankings by advocating for a more equitable and robust evaluation system by balancing diverse metrics and offering more standardized measures.

## 1 Introduction

University rankings play a pivotal role in shaping perceptions, influencing academic choices, and driving institutional policies. The international academic landscape is dominated by several influential ranking systems, each with its own distinct methodology and criteria. These include the Times Higher Education (THE) World University Rankings, Quacquarelli Symonds (QS) World University Rankings, Academic Ranking of World Universities (ARWU), often called the ShanghaiRanking, and US News Best Global Universities.

Despite the widespread use and influence of the current rankings, there are growing concerns about their methodologies and their inherent biases. Critiques often point to a reliance on reputation surveys, which introduces biases and favors universities from certain countries over the others (Toutkoushian and Webber, 2011; Shin, 2011; Safón, 2019; Bellantuono et al., 2022). Using surveys can skew rankings and does not necessarily reflect the true quality or impact of the educational institutions being assessed (Tomar, 2023). Even the ARWU methodology, which omits surveys and relies on arguably more objective metrics like the number of papers published in Nature and Science, has been shown to contain its own biases (Safón and Docampo, 2020).

A second pitfall in the current methodologies is the usage of article-level citation-based metrics. In general citations suffer from the Mathew Effect or what is known as “the rich gets richer, and the poor gets poorer.” When it comes to citations, this implies that a highly cited paper is more likely to get cited compared to other papers (Yang et al., 2015). Citation metrics can be easily gamed using various methods (Ioannidis and Maniadis, 2024). This had led to the rise of “citation cartels” which are groups of people who artificially boost their citations by citing each other, mostly in irrelevant contexts (Catanzaro, 2024). Another issue is the phenomenon of “hidden citations,” where foundational knowledge is used without direct attribution, potentially underestimating the impact of original research. In fact, important discoveries tend to have more hidden than actual citations (Allen, 2024).

University rankings often fail to account for the diverse missions and strengths of universities, particularly those focusing on regional development, innovation, or specialized programs that do not attract international students or faculty (Sauder and Espeland, 2009; Rauhvargers, 2013). In the past few years, many reputable universities have decided to withdraw from university rankings. Most notably, multiple top law and medicine schools have withdrawn from the U.S. News Rankings (CollegeNET, Inc., 2023), including Yale Law School which ranked first in every year the ranking was released (Yale Law School, 2022). Universities who withdrew from rankings stated various flaws in their methodology. For example, the University of Utrecht stated highly questionable data and methods by all makers of the rankings (Utrecht University, 2023), while the University of Zurich stated that current rankings focus on output rather than quality (UZH News, 2024). With increasing focus on academic rankings, it is crucial to address setbacks and biases present in ranking data (Chang and Liu, 2019).

This paper introduces a new set of metrics designed to focus on research quality rather than quantity. In particular, we propose using journal citation-based metrics and overall scholarly output as alternative indicators of research performance, rather than relying on article-level citation metrics. Journal citation-based metrics consider the aggregate citations received by all articles published in a journal, offering a broader perspective on a journal's impact. For instance, a journal's CiteScore is calculated by dividing the total number of citations received by a journal over the past four years by the total number of Scopus-indexed documents of the same type published during the same period. This approach provides a more comprehensive evaluation of research influence by reflecting the overall impact of a journal rather than the variability of individual article citations. The metric could also be used for assessing the research performance of academic institutions as it has a high correlation with the current ranking schemes. While our proposed method is not without limitations, it addresses many biases inherent in current assessment approaches.

## 2 Current ranking systems

### 2.1 Times higher education (THE)

THE rankings are well-regarded for their comprehensive approach, which assesses universities across five key dimensions: teaching, research, citations, international outlook, and industry income. Despite their broad analytical scope, THE rankings have been criticized for heavily weighting reputation surveys, which can subjectively benefit more established, well-known institutions, especially those in English-speaking regions. Moreover, since reputation surveys are sent to academicians nominated by the university itself, they can give a misleading image. For instance, a university can choose to only send the survey to people working in a field of study the university is known for. This could lead to a high reputation score although the university might not hold the same reputation in other fields. The emphasis on reputation often overshadows objective measures of educational quality and output (Marginson, 2014; Hazelkorn, 2015).

### 2.2 Quacquarelli symonds (QS) World University Rankings

Similar to THE, QS rankings rely significantly on reputational surveys, with them contributing to around half of the overall score through academic and employer reputation metrics. As mentioned above, these surveys introduce bias toward some universities and countries. QS ranking also includes a faculty to student ratio and internationalization metrics which can skew results in favor of institutions that perform well in these areas but may not necessarily excel in delivering quality education (Hazelkorn, 2015; Postiglione and Jung, 2017). Internationalization metrics can be inherently biased toward universities in developed countries. This bias arises because these institutions often have the resources and reputation to attract international faculty from developing countries, while the opposite is less common.

### 2.3 ShanghaiRanking (ARWU)

Unlike THE and QS rankings, ShanghaiRanking does not use reputation surveys. Instead, it places a stronger emphasis on research output and quality, including indicators such as Nobel Prizes and Fields Medals won by alumni and faculty, and the number of highly cited researchers. This favors a small percentage of older, more established universities that have had a longer time to accrue prestigious awards and notable alumni (Liu and Cheng, 2005; Karakhanyan and Stensaker, 2020). Namely, out of the 1,000 ranked universities in ARWU 2024, 76.6% had an alumni score of zero while 84.7% had an award score of zero. ARWU goes beyond looking at scholarly output in general, it places an emphasis on the output's quality by giving high weight to papers published in Nature and Science (N&S). Although the emphasis on output quality is a step in the right direction, only including papers in N&S gives an advantage to universities with good health and life sciences research quality while not rewarding universities with good research quality in other fields. For instance, the University of California San Francisco (UCSF) ranked 20th worldwide in the 2023 ARWU ranking despite having only health and life sciences programs and having an alumni score of zero. Their ranking is largely due to their N&S score, which is in the Top 9 worldwide. In cases where the universities are specialized in humanities and social sciences, ARWU mentions that the N&S weight is relocated to the other indicators, not to an indicator reflecting research quality. The N&S metric is also criticized for leading to biases (Safón and Docampo, 2020).

## 3 Materials and methods

The presented model aims to maximize the correlation between the selected set of metrics and the ShanghaiRanking scores. Given that the ShanghaiRankings are widely regarded as more robust and research-centric than other global university rankings, we initially use them as the target variable for correlation analysis with our research metrics. Subsequently, we extend our analysis by correlating these metrics with THE and QS rankings for a broader perspective. We utilize Orthogonal Matching Pursuit (OMP) for feature selection and GridSearchCV for optimization. To identify the most influential factors, we select the feature set demonstrating the highest correlation with the ShanghaiRanking score. Subsequently, we assign weights for the selected features through a linear programming approach to achieve the highest possible correlation with the ShanghaiRanking scores.

The following metrics (features) are selected by OMP: Scholarly Output, Output in Top 5% Citation Percentiles (%), Publications in Top 1% Journal Percentiles by CiteScore Percentile (%), Publications in Top 1% Journal Percentiles by SNIP (%), and N&S.

The optimization method determines the weight that should be assigned to each metric. We first normalize the metrics by dividing each metric by its maximum value, thereby scaling the range of each feature to [0, 1]. The normalization ensures that each feature contributes equally to the prediction, avoiding bias toward features with inherently larger scales.

Let *x* denote the matrix of features and *y* the vector of the target variable. The objective function aims to minimize the sum of the differences between the predicted values and the actual values of the target variable. This minimization problem can be formally formulated as follows:


$$\begin{array}{l} \text{Minimize}\overset{n}{\underset{i=1}{\sum }}{\left(y_{i}-\underset{j\in F}{\sum }w_{j}x_{ij}\right)}^{2}, \end{array}$$

where


$$\begin{array}{l} 0\leq w_{j}\leq 1,\;\forall j\in F\\ \underset{j}{\sum }w_{j}=1,\;\forall j\in F. \end{array}$$

The five selected features are represented in the set *F* = {1, 2, 3, 4, 5}. The target variable is assumed to be in the last column of the dataset.

We compute the proposed metrics for the Top 500 universities featured in ARWU. The remaining data are collected from SciVal and Scopus. All metrics we use are listed below.

*SciVal metrics:*

- Scholarly output.
- Output in top citation percentiles (1%, 5%, 10%, 25%).
- Field weighted citation impact.
- Publications in top journal percentiles by CiteScore (1%, 5%, 10%, 25%),
- Publications in top 1% journal percentiles by SNIP (1%, 5%, 10%, 25%),
- Publications in top journal percentiles by SJR (1%, 5%, 10%, 25%).

*ShanghaiRanking metrics:*

- Alumni: measures the number of alumni of an institution winning Nobel Prizes or Fields Medals.
- Award: the number of faculty and staff from an institution with a Nobel Prize or Fields Medal.
- HiCi: the number of highly cited researchers (as per Clarivate) whose primary affiliation is the institution in question.
- N&S: evaluates the number of papers published in Nature and Science index journals. Different weights are given based on the authorship position.
- PUB: represents the number of papers published in journals in the Science Citation Index-Expanded and Social Science Citation Index.
- PCP: stands for Per Capita Performance; a calculation of the weighted scores of the above five indicators divided by the number of full-time academic staff.

## 4 Results

The optimization process results in the following distribution of weights across the selected metrics:

- Nature and science publications (N&S): 30%.
- Scholarly output: 25%.
- Output in top 5% citation percentiles: 20%.
- Publications in top 1% journal percentiles by CiteScore percentile: 15%.
- Publications in top 1% journal percentiles by SNIP: 10%.

We calculate the correlation score of the metric with the Shanghai, QS, and THE rankings for years 2022 and 2023. Citation-based metrics such as the Field Weighted Citation Impact (FWCI) and Citations per Faculty are also included. Citations per faculty are calculated by dividing the total number of citations received by an institution within a broad academic category by the number of its faculty members. The Field-Weighted Citation Impact (FWCI) compares the citations received by an author or institution to the global average in the same field; a value of 1 indicates performance equal to the global average. Obviously, a paper could count multiple times by meeting multiple criteria (for example, publication in a N&S journal that is in the Top 1%). The obtained results are presented in Table 1. Typically, a correlation value between 0.10 and 0.39 indicates a weak correlation, a value between 0.40 and 0.69 suggests a moderate correlation, while a value between 0.70 and 0.89 signifies a strong correlation (Schober et al., 2018).

**Table 1:** Correlation of different metrics with the overall university ranking.

	**Shanghai 2023**	**Shanghai 2022**	**THE 2023**	**THE 2022**	**QS 2023**	**QS 2022**
FWCI (excl. self-citations)	0.364	0.364	0.409	0.462	0.348	0.337
Citations per faculty	0.375	0.381	0.431	0.363	0.375	0.375
SciVal metrics^*a*^	0.897	0.819	0.854	0.861	0.700	0.704
Journal quality + Scholarly output	0.854	0.811	0.819	0.837	0.695	0.689

^a^The selected metrics listed above without the journal quality.

SciVal metrics demonstrate the strongest correlation with overall university rankings. Interestingly, citation-based metrics show only weak to moderate correlations across all ranking systems and years. For example, the FWCI and Citations per Faculty metrics displayed correlation values ranging from 0.33 to 0.46. These similar correlation levels are expected due to the inherent positive relationship between the FWCI and the total number of citations. While the proposed metric strongly correlates with THE and ARWU rankings, its correlation with QS is slightly weaker. This is likely because 80% of the QS ranking weight is not directly related to research output or citation metrics, making it more challenging to predict using this approach.

We have also achieved strong correlation with the journal quality and scholarly output only approach, significantly higher than the FWCI and Citations per Faculty metrics. In fact, excluding other components has almost no effect on the correlation with Shanghai 2022, QS 2022, and QS 2023 indicating that focusing on journal quality only could be the way forward. While we considered removing scholarly output from our metric, this approach would unduly favor institutions with limited publication volume concentrated in the top 1% of journals. For instance, an institution with a handful of publications in top-tier journals could achieve a higher score than one with hundreds of publications in the top 5% (but not the top 1%). This disparity arises because the total volume of scholarly output is not factored in, potentially misrepresenting the overall research productivity of institutions.

In general, the results indicate that incorporating journal quality metrics with precisely tuned weights, can significantly enhance the accuracy of predicting university rankings. The success of the optimized weights in achieving a high correlation with the different ranking schemes suggests that universities could potentially focus on these areas to improve their performance in global rankings.

## 5 Discussion

### 5.1 Implications

Enhanced focus on quality metrics: institutions striving for higher global rankings might consider investing more strategically in the areas highlighted by the presented model, such as enhancing the quality and impact of their research outputs, especially in high-impact journals. Improving research quality can be fostered through strategic adjustments to hiring and promotion processes, with increased emphasis placed on publications in top-tier journals. Furthermore, encouraging interdisciplinary research can elevate scholarship in less-cited fields like mathematics. Notably, our analysis revealed a weak correlation between article-level citation metrics (citations per faculty and FWCI) and university rankings, suggesting that these metrics should be de-emphasized in evaluation processes.

Data-driven decision making: the methodology being used for developing this ranking system provides a framework for universities and educational policymakers to apply similar approaches to other aspects of educational assessment and strategy planning.

Reform in ranking systems: insights from the obtained results could serve as a basis for reforming how global university rankings are computed, advocating for a more balanced approach that fairly represents the diverse strengths of institutions across different regions and disciplines.

### 5.2 Limitations

While the results are promising, the study acknowledges certain limitations that should be addressed in future research:

*Citation limitations:* while citation count is susceptible to manipulation and may not accurately reflect the true value of a study, it is often used as a proxy for research impact. The proposed metrics assigns equal weight to papers published in the same venue, year, and field, regardless of citation count. Citation cartels could also increase a journal's ranking, but this is much less common as it is harder to achieve for top journals and easier to solve by simply excluding the journal from the evaluation.

*Journal rankings:* although the vast majority of reputable journals are ranked, some good journals in subjects like humanities may not be ranked. Moreover, journals in foreign languages tend to be underrepresented.

*Expansion to other universities:* this study did not explore whether the proposed metric still correlates with the ranking scores of universities outside the Top 500. This should be explored in future endeavors as ranking schemes tend to have a smaller intersection as we go deeper.

## 6 Conclusion

This paper presents a new ranking metric tailored to address the limitations of the citation-based metrics observed in existing global university ranking systems, such as THE and QS World University Rankings. By strategically weighting various metrics, we have developed a novel framework that strongly correlates with established university ranking models. Unlike these models, which often incorporate subjective reputation surveys and article-level citations, our approach prioritizes objective measures of research quality and utilizes journal-level citations for a more comprehensive assessment. High correlation was achieved with all ranking schemes, the highest being with the 2023 ShanghaiRankings (0.897). This indicates the effectiveness of the presented model in capturing the key performance indicators that influence global university rankings. It also highlights the potential shortcomings in current ranking methodologies that might overemphasize or under-represent certain aspects of university performance. The findings from this study have several implications for educational policy and institutional strategy. Future research could explore subject-specific rankings, employing predefined lists of venues to assess research quality within particular disciplines. Additionally, developing objective methods for evaluating non-research universities presents a promising avenue for further investigation.

## Data Availability

Publicly available datasets were analyzed in this study. Data was extracted from SciVal.
